# Socioeconomic Appraisal of an Early Prevention System against Toxic Conditions in Mussel Aquaculture

**DOI:** 10.3390/ani12202832

**Published:** 2022-10-19

**Authors:** Athanasios Ragkos, Dimitrios Skordos, Georgia Koutouzidou, Ioannis A. Giantsis, Georgios Delis, Alexandros Theodoridis

**Affiliations:** 1 Agricultural Economics Research Institute, Hellenic Agricultural Organization—DIMITRA, Kourtidou 56-58, 111 45 Athens, Greece; 2 Institute of Plant Breeding and Genetic Resources, Hellenic Agricultural Organization—DIMITRA, 57001 Thessaloniki, Greece; 3 Department of Animal Science, Faculty of Agricultural Sciences, University of Western Macedonia, 53100 Florina, Greece; 4 School of Veterinary Medicine, Aristotle University of Thessaloniki, 54124 Thessaloniki, Greece

**Keywords:** cost–benefit analysis, linear programming, risk management

## Abstract

**Simple Summary:**

In order to mitigate the destructive effects of the occurrence of toxic conditions on mussel farming, an automated early prevention system against such conditions was installed. The analysis in this paper demonstrates that the investment is highly profitable and can contribute to achieving broader socioeconomic benefits at the local and regional level.

**Abstract:**

This paper examines the financial viability and potential socioeconomic effects of introducing and operating an automated, remote-controlled management system for mussel farms which uses probes of temperature, dissolved oxygen, and conductivity associated with prediction software to demonstrate the potential need for mussel movement between marine areas. This system provides an early warning to farmers regarding the presence of toxins in aquatic ecosystems, thus contributing to saving mussel production and avoidikng significant economic losses. The analysis combines two established methodological tools in agricultural economics (linear programming and cost-benefit analysis) and provides estimates of the Net Present Value of the investment under two scenarios—one reflecting the existing situation and one a possible future situation where the mussel production system is expanded. The results of the analysis reveal the mid- and long-term effects of using the automated system, both of which demonstrate that the system is economically viable even if it contributes to saving mussel production from toxicity occurrence for only one year during its period of operation. The annual gross margin in the first scenario was €386,069 but almost tripled in the second scenario (€1,154,649). In addition, the future development and expansion of the mussel sector will likely be based on larger farms with an entrepreneurial and exporting orientation where risk mitigation systems, such as the one appraised in this paper, can play an important role.

## 1. Introduction

Mussel (*Mytilus galloprovincialis*) farming is an activity of specific importance to mainland coastal areas of Greece, as it is the source of supplementary (or main) incomes for families involved in the sector. Previous research [1,2,3] showed that this form of aquaculture involves a relatively small number of heterogeneous farms and producers, with different patterns of entrepreneurial organization and production practices. Mussel farms in Greece mainly apply the longline system in which mussels are reared in mussel sleeves suspended by a backline rope attached on buoys on the sea surface until they reach the desired commercial size. Since mussel farming does not have high variable costs (as no feeding costs are required because mussels are filter-feeder organisms consuming organic material that they ingest from seawater), it is not subject to the development barriers which are common to other primary production sectors (e.g., limited liquidity and high prices of inputs) and is, therefore, expected to demonstrate high resilience under crises.

The performance and future prospects of the mussel sector in Greece are largely dependent on environmental conditions—including the physiochemical properties of water—and possibilities for organized genetic improvement schemes that would increase productivity are still low. As Avdelas et al. [4] pointed out, the decline of the EU mussel sector can be attributed mostly to environmental and less to socioeconomic reasons. A particularly common external threat relates to unpredicted changes in water properties such as temperature and dissolved oxygen [5]. Especially over the last few years, climate conditions have become increasingly volatile, and high water temperatures during summer are responsible for high mortality rates of mussels as well as thermal and oxidative stress that may decrease productivity and growth rates [6]. High water temperatures combined with dissolved oxygen concentration beyond specific limits may also be responsible for the increased concentration of biotoxins or toxic phytoplankton [7] as well as for high populations of the protozoan *Marteilia refrigens* which is the most harmful mussel pathogen [8]. Toxic bacteria concentrations in marine ecosystems are also increasing due to industrial pollution, but mainly where agricultural intensification is pursued through the use of phosphate fertilizers [9,10].

The presence of toxins in aquatic ecosystems, therefore, generates important threats for the viability of mussel farms as well as for public health. Indeed, mussels are distributed for human consumption after costly processing in sanitation facilities, while they may be discarded—or their production area may even be put out of operation—if toxins are found in high concentrations. Greek mussel production zones are divided into three categories (Zone A, B, or C) according to the presence of bacterial toxins within bivalve tissues. Most of the areas are categorized as B, which means that mussels are treated in sanitation facilities before commercialization, in order to safeguard public health. In fact, in cases where the results of tests on the microbial load of the mussels and the presence of toxic phytoplankton indicate that live mussels may be dangerous for human health, competent authorities can “seal” the production zone until the permissible sanitary conditions are restored. In addition, under toxic conditions, dead mussels increase toxicity which also renders the mussels of other farms in the area non-commercial, thus leading to 100% production losses.

These destructive implications can be avoided if adverse environmental conditions are detected at a very early stage and mussels can thus be moved to another part of the sea area within the same basin, where conditions are better. The SmartMussel project proposes a model of automated, remote-controlled management system for mussel farms, which uses probes of temperature, dissolved oxygen, and conductivity associated with prediction software to demonstrate the potential need for mussel movement between marine areas. A detailed description of the development and operation of the SmartMussel system is provided by Georgoulis et al. [5]. An experimental setting of the system was installed in 2020 in the area of Vistonikos Gulf (Northeastern Greece). The system consisted of two different lines of rigs and three groups of sensors installed at different depths, all of which measure temperature, dissolved oxygen, and conductivity in real time and with very high accuracy (dissolved oxygen at least 0.2 mg/L; temperature ±0.1 °C; conductivity at least 1%). The three groups were connected wirelessly and transmitted data through a telematics system using a cell phone network. The system was also equipped with an automatic alarm system installed in case critical thresholds were exceeded because of thermal stress in summer or winter. In case of such toxicity prevalence, mussel farmers were informed before their production was destroyed and had the necessary time to move them to another part of the sea where conditions were better or to harvest earlier. The system was constructed using materials which were suitable for aquatic conditions and sensors and other components of small size compatible with the size of mussel floats.

As part of the SmartMussel project, this paper examines the potential socioeconomic effects of introducing and operating this automated system. In particular, the analysis examines the economic performance of investing in this system under two scenarios, one which optimizes the current situation and one depicting a future optimal situation where the production system is developed and expanded. Through this analysis, the mid- and long-term effects of using the automated systems are approached, thus demonstrating the necessity of introducing it and its usability in terms of risk management.

The introduction of new technologies to assist the development of mussel farming has been in the spotlight of recent research. A prime example is the study of Cebu [11], who examined the effects of the implementation of the Bamboo Tray Module on mussel production volumes. He found that this innovation outranked the performance of traditional farming methods, increasing productivity by 50%. Coelho-Caro et al. [12] presented an automatic mussel classifier system which—based on machine learning tools—managed to achieve a recognition rate of 95% among five mussel species and different sizes. Martín-Rodríguez et al. [13] presented a method by means of which Sentinel 2 images were used in order to perform a census of mussels grown on platforms. Borcherding [14] presented the Dreissena-Monitor which served as a biological warning system for aquatic conditions. The system is based on the development of an algorithm measuring the behavior of mussels under normal conditions against behavior after exposure to toxicity. The system was found to have numerous advantages in terms of easiness and reliability. Shen and Nugegoda [15] described the development of a microcontroller-based device for the real-time biomonitoring of mussel behavior which assesses the behavior against that of exposure to environmental contaminants. Another example is the work of Montella et al. [16], who described the way that a workflow engine, based on Galaxy and Globus technologies, can be used to predict the impact of pollutants in mussels farming areas and, consequently, on the quality and quantity of the products. Although these studies describe promising technologies which contribute to more effective management and/or reduction of risks and related losses, they lack the element of socioeconomic assessment. Our study presents how the combination of two established methodological tools in agricultural economics (linear programming [LP] and cost–benefit analysis [CBA]) can be used to fill in this gap. To our knowledge, this is the first paper approaching the socioeconomic appraisal of technological advancements in mussel farming in this way.

A few studies have examined the economic performance and competitiveness of mussel farms. Some examples include the papers of Nguyen et al. [17], who assessed the financial feasibility of blue mussel (*Mytilus edulis*) farms in the Great Belt (Denmark), and Theodorou et al. [18], who investigated the profitability of 49 mussel farms in Greece. Both studies found that large-sized farms with low- labor intensity can achieve economies of scale and therefore can be more profitable than smaller ones. This finding is also confirmed by Gren and Tirkaso [19], who estimated the cost of producing mussels by implementing a meta-regression analysis. In addition, the profitability of mixed mussel farming systems has also been investigated (e.g., [20,21]). The impact of environmental factors on the production of mussel farms is a topic that also received much attention in the last decade (e.g., [5,22,23]).

## 2. Materials and Methods

### 2.1. The Study Area

**Figure 1:**
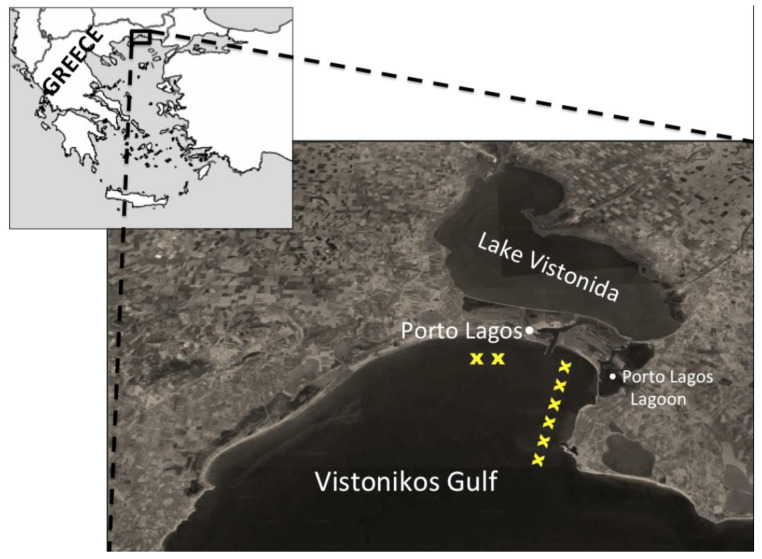
Satellite image map showing the study area. Mussel farms’ locations are indicated with yellow “x”. There is a possible area of expansion of the mussel farming area towards the southeastern direction.

Porto Lagos in Vistonikos Gulf was chosen as the mussel farming area for the pilot application of the SmartMussel system (Figure 1). The area is situated in the Region of Thraki in Northeastern Greece. There are 8 mussel farming units operating in the area, which are new, more sparse (longer distances between the different units), while they have faced problems in the past with regard to the high concentration of toxic substances as a result of which mussel commercialization was prohibited for certain periods. For example, in the spring of 2010, excessive growth of phytoplankton due to environmental factors led to complete destruction of the mussel production in the area. In addition, mussel farms in the area are greatly influenced by the flow of marine currents. For all these reasons, the farms located in the area were an ideal site to test the efficiency of the proposed system.

### 2.2. Data

The analysis is based on data from a survey of local mussel farms in the study area. The survey was conducted with in-person interviews in 2020–2021 with all eight local mussel farmers (i.e., the whole population was interviewed). Data collected during the survey were used in order to calculate technical and economic indicators of farm management. However, due to the small number of survey participants, the indicators that were thus calculated were re-assessed and calibrated based on data and results from previous research in Thermaikos Gulf (Northern Greece), which is a neighboring area of high importance for Greek mussel aquaculture [2,3]. These data were from previous years; thus, the final set of indicators incorporates possible fluctuations and price volatility.

The final set of indicators thus calculated was used to depict the profile of three “typical” mussel farms, each one of which corresponds to a farm type which is representative of the farm typologies in the study area according to size (i.e., the occupied sea surface area). Following to the information reported in Table 1, Small Farm (SF) occupies only 0.65 ha of sea area and produces 39,000 kg of mussels each year. It is predominantly family-owned and managed, and its main role is to supplement incomes from other activities. Medium Farm (MF) occupies 1.40 ha of sea surface area and produces around 90,700 kg of mussels per year on average. Its main characteristic is effective labor organization, and while it is also family-owned, hired workers assume significant roles where needed. Large Farm (LF), on the other hand, is considerably more extended (2.80 ha of sea surface area) and produces significantly higher quantities of mussels (190,000 kg). Due to their size, their labor requirements are more than twice those of MF, and they thus rely on hired labor.

Prices differ across the three typical farms, and LF especially achieves considerably higher prices than the other two, mainly because its high volume of production allows them better bargaining and access to markets. Indeed, while SF sells mainly to local restaurants and retailers and MF targets local and regional markets, LF is able to approach nationwide commercial agents and exporters. Differences in prices and productivity are reflected in differences in the gross revenue of the three farms, ranging from €15,513 for SF to almost six times higher for LF (€75,120). Although production costs are also significantly higher for LF (€59,233), this farm is the most profitable, while for MF revenues marginally exceed expenses, and for SF expenses are 36% higher than revenues. These differences are also due to the organization of labor in SF and the efficient use of fixed capital by LF.

### 2.3. Methodological Approach

The methodological approach of the analysis combines a LP model [24,25] with a CBA. In particular, an LP model is constructed where the three types of mussel farms (SF, MF, and LF) are included as variables and relevant constraints, and the optimal solution shows the number of farms from each type that maximize the economic output (gross margin).

The basic LP model matrix is presented in Table 2, where:

- The objective function (which is maximized in the optimization problem) denotes the gross margin achieved by each typical farm (GM_SF, GM_MF, and GM_LF), while the optimal solution shows the number of farms that belong to each type which achieves the maximum gross margin.
- Constraints that relate to
  ○ The available sea surface (ASS) and the sea surface that can be occupied by each typical farm (SS_SF, SS_MF, and SS_LF)
  ○ The available family labor (AFL) and the family labor required by each typical farm (RFL_SF, RFL_MF, and RFL_LF)
  ○ The available hired labor (AHL) and the family labor required by each typical farm (RHL_SF, RHL_MF, and RHL_LF)
  ○ Variable capital available to the mussel farms in the area (VC) and their requirements per farm (VC_SF, VC_MF, and VC_LF)
- Mussel production per farm type (Prod_SF, Prod_MF, and Prod_LF)

**Table 2:** Linear programming matrix.

	SF	MF	LF	HLAB	PROD
*Objective function (Max)*	GM_SF	GM_MF	GM_LF	-HLHR	0
*ASS>=*	SS_SF	SS_MF	SS_LF		
*AFL>=*	RFL_SF	RFL_MF	RFL_LF		
*0>=*	RHL_SF	RHL_MF	RHL_LF	−1	
*AHL>=*				1	
*VC>=*	VC_SF	VC_MF	VC_LF		
*0>=*	Prod_SF	Prod_MF	Prod_LF		

The results of the model indicated the optimal structure of the mussel sector, which highlighted how each farm type could be viable compared to other types. In order to understand sectoral dynamics, however, the methodological approach examined two scenarios. The first (“Current” scenario) illustrated the status-quo situation where the three types of mussel farms under examination coexist in the same area. This scenario referred to the short run and it was implicitly assumed that the existing system organization could not be changed substantially. The second scenario (“Future”) reflected a possible mid-term situation in which mussel farming in the study area would be expanded. This expansion is subject to administration issues related to marine spatial planning, as it requires that the area is established as an “Area of Organized Development of Aquaculture” (acronym in Greek is “POAY”). This adjustment could increase the area available to mussel farmers up to 120 ha (currently it is close to 45 ha).

The results of the LP model were then used within a CBA framework, which was related to the number of possible outbreaks of adverse conditions that could lead to the destruction of production. The CBA was performed for both scenarios, i.e., once for “Current” and once for “Future”. The basic data that were used for the CBA are reported in Table 3. Although the basis of the system could have a longer productive life, it was considered that after 12 years the system would be technologically obsolete and could be replaced by more modern equipment. Therefore, the investment was appraised for a period of 12 years. The discount rate was 4% in the “Current” scenario and 6% in the “Future”, because the latter was linked with higher uncertainty (related to resolution of space issues, also mentioned as a weakness by Avdelas et al. [4]). The initial investment was estimated at €30,000 (based on actual costs incurred for the full establishment and initial testing of the system), while the residual value was estimated at 10% of initial investment costs. Annual costs included basic maintenance costs estimated as 6% of initial investments costs, while in year 6 mechanical equipment would be renewed (20% of investment costs in addition). Annual benefits corresponded to the gross margin that was calculated by means of the LP model, since the occurrence of toxic conditions entailed the full loss of all mussel production in the area. Therefore, annual benefits were estimated as the income (gross margin) that is not lost due to timely prevention. The CBA took into account the probability that toxic conditions may occur in one or multiple years, and the Net Present Value (NPV) of the investment was calculated separately under the assumption that production losses were avoided for 0 (i.e., no occurrence of toxic conditions) to 12 years (toxic conditions every year for the whole duration of the investment).

## 3. Results

### Results of the LP Model

Table 4 presents the results of the LP model for both scenarios. In the “Current” scenario, the optimal structure of the mussel farming system includes 23 mussel farms, 50% of which are LF. MF and SF still exist, but they contribute only a small part of the total mussel production, which exceeds 3000 tons. The sector employs 49 persons full-time, almost half of which are hired workers; the high contribution of hired labor is due to the increase in the number of LF. The total gross margin slightly exceeds €386,000, and variable capital is around €460,000.

The “Future” scenario, however, depicts a different optimal situation for the sector. Since time allows a structural adjustment, it seems that the extended sea area is now occupied only by 43 LF in total, ruling out completely SF and MF. This development has important implications in the socioeconomic conditions in the sector. Total income (gross margin) is 3 times higher than the “Current” scenario (€1,154,649), but the use of variable capital is also 2.5 times higher (€1,152,000), which implies more intensive use of capital (fixed capital investments but also energy and other variable costs). Furthermore, the expansion of the sea surface area increases significantly jobs in the sector (120 persons full-time) with a considerable increase in family labor (58 persons against 24 persons in the “Current” scenario), which implies that additional employment opportunities will be provided to families interested to invest in the sector.

**Figure 2:**
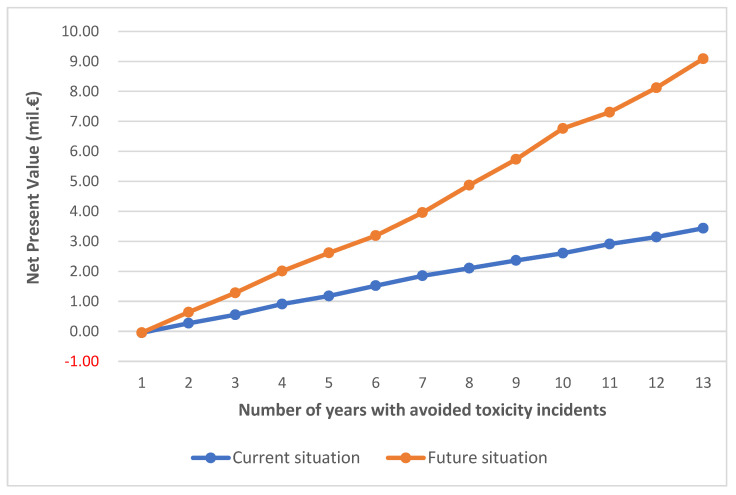
NPV of the investment on the installation of the SmartMussel system under different number of years of toxicity incident avoidance.

Based on the results of the LP model, the CBA of the investment was performed, and the results are reported in Figure 2. Apparently, as the number of years with avoided toxicity incidences increases, the NPV of the investment increases as well. The “Future” scenario performs much better than “Current”; however, the slope of the NPV curve in “Future” is much steeper, indicating that each incident avoided has a much more significant impact compared to “Current”. It is very important to notice that the NPV of the investment is positive even if only one incident is avoided. This demonstrates the importance of the system for the operation and sustainability of the mussel farming system in the area.

## 4. Discussion

The CBA shows that the establishment of the SmartMussel system is beneficial for the mussel farms in both scenarios. Although the NPV is positive for all cases under consideration, the important aspect is that the automated preventive warning system provides assurance to farms that leads to risk mitigation. It should be noted that toxicity incidences are not rare for Greek Mediterranean conditions, and especially under the adverse climate dynamics, it can be expected that their occurrence will increase in the following years. Therefore, the SmartMussel system provides an effective risk mitigation tool for farmers already in the sector or for those who could engage with it in a scenario like the “Future” one.

The results of the LP model confirm the socioeconomic importance of mussel farming. In both scenarios there are significant gains in terms of gross margin. The prevalence of LF in the “Future” scenario demonstrates how these farms—which are larger in size and achieve higher profitability—are able to expand over other farm types if external (e.g., administrative) conditions are favorable. The same profile of farms was depicted by Theodoridis et al. [2], who showed that these farms are more efficient than smaller ones. Also, Theodoridis et al. [3] found that mussel farms adopting “best practices” were larger and took advantage of this to organize labor and to use fixed and variable capital more efficiently, thus reducing their total production costs. A mussel sector consisting mainly of such farms would thus be more viable in the long run and the results of our current study demonstrate that risk reduction due to the operation of the SmartMussel system can boost its performance.

Even more important, however, is the fact that significant improvements are witnessed in terms of employment. The profile of the typical farms is slightly different in terms of the family labor/hired labor ratio compared to previous studies (e.g., [2,3]), because in our study family labor plays a more important role in all three typical farms. This illustrates the importance of the activity for the social sustainability in the area. Indeed, mussel production has been declining in the past few years in Greece but also in Europe [22]. Successful implementation of the “Future” scenario could have important positive implications on social sustainability, stability, and cohesion at the local/regional level by ensuring more jobs and involving more families. As Theodorou et al. [18] pointed out, socioeconomic sustainability is linked to the emergence of larger farms but also to the development of cooperation schemes between farmers.

Although the “Future” scenario performs better than the “Current” one, an issue that could affect this success in the mid-term is product commercialization. According to European Market Observatory for Fisheries and Aquaculture Products [26] mussel production in Greece is negligible compared to big EU producers such as Spain—exports account for only 4% of total EU exports with declining trends—while production has also been declining from 2007 to 2016 in the country. In addition, even the LF in our study is of relatively lower size compared to the EU average, with turnover lower by 70% than what is achieved by longline mussel farms in the EU [22]. Under these conditions, the “Future” scenario could be proven unfeasible if better access to markets is not ensured for additional produce. Supply chain measures are required to boost the commercialization of Greek fresh mussels in general while also other ways to increase acceptability by consumers have been proposed, such as organic and origin certification [22].

One of the factors that need to be taken carefully under consideration is the level of effectiveness of the system, i.e., the degree to which the system will successfully provide early warnings about toxicity occurrence. Data from the implementation of the project during the period 2020–2021 showed that the system responds immediately supporting the prohibition of the negative effects caused by the seawater temperature rise at levels beyond 28 °C in August 2020. This approach can be used complementary to other methods and systems, for instance with studies of behavioral monitoring, as described by Shen and Nugegoda [15]. Since socioeconomic appraisals for such systems are not generally available in the literature, integrated assessments (either comparative or supplementary) of the two systems could demonstrate their effectiveness.

The model considers that the production practices of the three types of farms remain the same in the two scenarios. This could pose a limitation, as especially in the “Future” scenario it is possible that basic parameters such as product prices and productivity could change under pressures from the external environment. However, the analysis has shown that the expected benefits exceed by far the cost of the system, and such changes could alter the slope of the NPV curves (like the ones in Figure 1) but still the NPV would be high and positive even with limited toxicity occurrence incidents.

## 5. Conclusions

This paper employed a combination of LP and CBA in order to propose a methodology for the socioeconomic appraisal of innovations in mussel aquaculture. By developing two scenarios, the economic and social effects of introducing a system for timely prevention in cases of occurrence of toxic environmental conditions were captured and analyzed. Through this analysis, the mid- and long-term effects of using the automated systems were approached, thus demonstrating the necessity of introducing it and its usability in terms of risk management. Among other findings of the analysis, the contribution of the sector to employment and specific proposals for increasing the viability of each farm type were revealed. The results of this paper show that there is ample room for introducing technological innovations in mussel aquaculture in Greece, since they could contribute substantially to taking advantage of its development prospects. Despite the fact that this development is subject to administrative barriers and to adverse environmental conditions, the dynamics of the sector could benefit from improved risk management. If strategically organized, an integrated plan for the development of the sector could bring significant social and economic benefits which could be supported by technological innovations like the SmartMussel system, which is low-cost and relatively easy to use and maintain. More research is required, however, with regards to the organization of the system—especially in the “Future” scenario—in terms of cooperation among farms as well as the incurrence of the costs of installation and maintenance and operation of more risk mitigation systems. Participatory methods are needed in order to capture the aspirations of SF, MF, and LF farmers and to design effective governance schemes that will provide for a fair allocation of costs across farms.

## Figures and Tables

**Table 1:** Technical and economic indicators of typical mussel farms.

	Small Farms (SF)	Medium Farm (MF)	Large Farms (LF)
Average acreage (ha)	0.65	1.40	2.80
Production (kg)	39,000	90,700	190,000
Price (€/kg)	0.362	0.353	0.381
**Labor (€)**	**2819**	**2273**	**4807**
Family (€)	1775	1232	2336
Hired (€)	1044	1041	2471
**Gross revenue (€)**	**15,513**	**35,994**	**75,120**
**Production expenses (€)**	**21,124**	**34,499**	**59,233**
Sea acreage (Rent) (€)	360	727	1468
Labor (€)	9710	8504	19,656
Capital (€)	11,054	25,268	38,109
Variable (€)	7831	16,350	26,880
Fixed (€)	3222	8918	11,229

**Table 3:** Cost–benefit analysis data and assumptions.

	Scenario 1 Current Situation	Scenario 2 Future Situation
**Investment costs (Year 0)**	**30,000 €**	**30,000 €**
Multiparameter measurement sensor for temperature, pressure, salinity and dissolved oxygen	11,000 €	11,000 €
Connector cables	2800 €	2800 €
Telemetric digital stand-alone datalogger	8000 €	8000 €
Solar panel	5400 €	5400 €
Labor costs	2800 €	2800 €
**Annual operation costs**	**1800 €**	**1800 €**
**Maintenance costs (replacement of equipment-Year 6)**	**6000 €**	**6000 €**
**Residual value**	**6000 €**	**6000 €**
**Years of productive life**	**12**	**12**
**Discount rate**	**4%**	**6%**

**Note:** Rows in bold correspond to basic cost categories and data directly used in the Cost-Benefit Analysis.

**Table 4:** Results of the LP model under the two different scenarios.

	Scenario 1 Current Situation	Scenario 2 Future Situation
**Number of farms**	**23**	**43**
**Sea area (ha)**	**45**	**120**
**Synthesis of farms**		
Large farms (LF)	50%	100%
Medium farms (MF)	30%	0%
Small farms (SF)	20%	0%
**Labor (full time persons)**	**49**	**120**
Family (full time persons)	25	58
Hired (full time persons)	24	62
**Variable capital (€)**	**459,774**	**1,152,000**
**Gross margin (€)**	**386,059**	**1,154,649**
**Mussel production (kg)**	**3,002,175**	**8,142,857**

## Data Availability

The data presented in this study are available on request from the corresponding author. The data are not publicly available because there will be a two-year period until the authors decide to publish additional papers.
